# Associations of social isolation with memory and cognitive function in middle-aged and older Chinese adults

**DOI:** 10.1007/s40520-025-02987-9

**Published:** 2025-03-08

**Authors:** Ting Feng, Rui Qiang Li, Lin Xu

**Affiliations:** 1 Johns Hopkins·Bloomberg·School·of·Public Health, Baltimore, USA; 2 School of Public Health, Sun Yat-sen University, 74 Zhongshan 2nd Road, Guangzhou, Guangdong Province China; 3 School of Public Health, The University of Hong Kong, Hong Kong, China; 4 Institute of Applied Health Research, University of Birmingham, Birmingham, UK; 5 Greater Bay Area Public Health Research Collaboration, Guangzhou, China

**Keywords:** Social isolation, Memory decline, Cognitive function, XGBoost, SHAP

## Abstract

**Background:**

Although social isolation has been identified as a risk factor for cognitive impairment, its potential impact relative to other documented risk factors has not been comprehensively quantified, leading to its underestimation in public health strategies. We aimed to address this gap by quantifying the contribution of social isolation to cognitive decline in the context of other risk factors.

**Methods:**

Social isolation was evaluated using a modified Social Network Index (SNI) and cognitive function through the Delayed Word Recall Test (DWRT) and the Mini-Mental State Examination (MMSE). Linear and logistic regression models were employed to analyze the associations between social isolation and cognitive outcomes, adjusting for demographic and health-related factors. Additionally, the XGBoost algorithm with SHapley Additive exPlanations (SHAP) was used to quantify the relative importance of predictors.

**Results:**

A total of 25,981 participants were recruited from 2003 to 2008. The mean age was 62.0 years, with 28.4% being men. Higher social isolation was significantly associated with lower DWRT (β=-0.15; 95% CI: -0.21 to -0.09) and MMSE scores (β=-0.34; 95% CI: -0.48 to -0.19), and higher odds of memory impairment (OR = 1.27; 95% CI: 1.15 to 1.40) and poor cognitive function (OR = 1.56; 95% CI: 1.23 to 1.99). XGBoost analysis ranked social isolation as the fifth most important predictor for MMSE scores (SHAP value = 0.175) and the eighth for memory impairment (SHAP value = 0.0133). Subgroup analyses indicated stronger associations among older adults, and individuals with lower education or manual occupation.

**Conclusion:**

Our findings showed that social isolation is an important risk factor for cognitive outcomes. This underscores the urgent need for targeted public health interventions addressing social isolation, alongside other key risk factors, to preserve cognitive health.

**Supplementary Information:**

The online version contains supplementary material available at 10.1007/s40520-025-02987-9.

## Introduction

With the rapid increase in the global aging population, the prevalence of cognitive decline and dementia has become an urgent public health concern, with projections showing a sharp rise in cases globally over the coming decades [1, 2]. Cognitive impairment not only reduces the quality of life for affected individuals but also places a significant burden on healthcare systems and caregivers. Among the various factors contributing to cognitive decline, social isolation garnered attention for its profoundly negative effects on cognitive health, particularly among older adults [3]. Studies have shown that social isolation is a critical risk factor for cognitive decline, as it often leads to reduced opportunities for cognitively stimulating social interactions, which are essential for maintaining cognitive function [4, 5]. The absence of social engagement may accelerate cognitive deterioration and increase the likelihood of developing conditions such as Alzheimer’s disease and dementia [6]. Furthermore, older adults with infrequent social interactions were found to be at a substantially higher risk of cognitive decline compared to those with more active social lives [7].

Despite the growing body of evidence linking social isolation to cognitive impairment, its relative impact in the context of other established risk factors, such as education, age and health behaviors, remains underexplored. This gap in understanding limits the ability to prioritize social isolation effectively within public health strategies aimed at mitigating cognitive decline. Additionally, cultural and social differences may influence how social isolation affects cognitive function in different populations, potentially modulating its impact. Addressing this research gap is crucial for developing more comprehensive interventions that consider the broader social determinants of cognitive health.

Therefore, this study aims to quantify the association between social isolation and cognitive function in middle-aged and elderly populations, with a particular focus on its impact on memory function. By integrating comprehensive socio-economic and health behavior data, this cross-sectional study provides robust evidence on the role of social isolation within the broader context of cognitive health, offering a theoretical foundation for future public health interventions targeting this modifiable risk factor.

## Materials and methods

### Study samples

The Guangzhou Biobank Cohort Study is a joint effort between the Guangzhou Twelfth People’s Hospital, the University of Hong Kong, and the University of Birmingham [8]. Participants were recruited from the “Guangzhou Health and Happiness Association for Respecting the Elderly” (GHHARE), a social welfare organization for individuals aged 50 and above. Between 2003 and 2008, GHHARE invited 32,850 members, and 30,430 (92.6%) consented to participate. GHHARE, which operates across ten branches in Guangzhou, had a participation rate of 7% among residents aged 50 or older. The questionnaire’s reliability and validity were assessed by re-interviewing 200 randomly selected participants six months into the study [8]. The study has been approved by the Guangzhou Medical Ethics Committee of the Chinese Medical Association, and all participants provided written informed consent before participation. In this study, we used the baseline data from GBCS conducted between 2003 and 2006, including 25,981 participants.

### Social isolation measurement

We identified four distinct categories of social contact using validation questions from the Berkman-Syme Social Network Index (SNI), with modifications tailored to the study context [9]. Given that in 2003, before the widespread adoption of smartphones, the Internet, and social media, telephone and email were the primary forms of non-face-to-face communication, email was included alongside telephone as a substitute method. The composite social isolation score was calculated by aggregating the scores across four types of social isolation: face-to-face contact with co-inhabitants, face-to-face contact with non-co-inhabitants, non-face-to-face contact (via telephone or mail), and club/organization contact, resulting in a total score between 0 and 7, where higher scores indicate greater degrees of social isolation [10]. To facilitate interpretation and allow for comparisons between distinct levels of social isolation, the composite score was further categorized into three groups: 0 (no social isolation), 1 (mild social isolation), and ≥ 2 (moderate to high social isolation).

### Memory function assessment

Cognitive function was evaluated using the Delayed Word Recall Test (DWRT) and the Mini-Mental State Examination (MMSE) [11, 12]. Delayed recall memory was assessed at both baseline (2003–2006) using the delayed 10-word recall test, as documented in previous GBCS studies [13]. Of the ten words utilized, four—“letter,” “ticket,” “grass,” and “arm”—were retained from the original English version of the test [12]. The words “pole,” “engine,” “cabin,” and “shore” were replaced with “corner,” “stick,” “book,” and “stone,” in accordance with the adapted Consortium 10-word list learning task [14]. To align the test with Chinese cultural contexts, “queen” and “butter” were substituted with “chairman” and “soy sauce.” Following a 5-minute distraction period in which participants answered other questions, they were asked to recall as many words as possible. This final recall was recorded as the DWRT score, ranging from 0 to 10. The total number of words recalled constituted the DWRT score. Memory impairment was defined as a DWRT score of less than 4 [15]. The MMSE was introduced during the second examination to further assess cognitive abilities. This 30-item test evaluates multiple cognitive domains, including orientation, memory, attention and calculation, recall, and language, with scores ranging from 0 to 30. Impaired cognitive function was defined as an MMSE score of less than 25 [16].

### Potential confounders

To account for potential confounders, we included a comprehensive set of demographic, lifestyle, and health-related variables known to influence both social isolation and cognitive outcomes. Age was categorized as < 65 or ≥ 65 years, and sex as men or women. Education level was stratified into three categories: primary or below, secondary, and college or above. Occupation was classified as manual, non-manual, or other, while personal annual income was divided into four categories: <10,000, 10,000–14,999, ≥ 15,000 Chinese Yuan, and not reported. Smoking status was classified as never, former, or current. Body mass index (BMI) was categorized as < 18.5, 18.5–24.9, 25.0–27.4, and ≥ 27.5 kg/m². Alcohol use was grouped as never, former, or current, and physical activity levels were categorized as inactive, moderate, or active. Self-rated health was categorized as good/very good or poor/very poor. Additionally, self-reported diabetes, hypertension, and dyslipidaemia were included as binary variables.

### Statistical analysis

In the descriptive analysis, we calculated frequencies for categorical variables and presented the mean, standard deviation, median, and interquartile range (IQR) for continuous variables. Wilcoxon rank-sum tests were used to compare continuous variables across levels of socioeconomic status, while chi-square tests were employed to analyze categorical variables. We examined the associations between the composite social isolation score and cognitive outcomes, including DWRT and MMSE scores. Linear regression models were used to estimate β coefficients and 95% confidence intervals (CIs) for DWRT and MMSE scores, while logistic regression models were applied to assess the odds ratios (ORs) and 95% CIs for memory impairment (DWRT < 4) and poor cognitive function (MMSE < 25). Both crude and adjusted models were used. The adjusted models controlled for potential confounders, including age, sex, education level, occupation, personal annual income, smoking, sleep duration, BMI, alcohol use, physical activity, self-rated health, and self-reported conditions (diabetes, hypertension, dyslipidaemia).

In addition, we applied the eXtreme Gradient Boosting (XGBoost) algorithm to assess the influence of various predictors on cognitive outcomes [17], specifically DWRT and MMSE scores, along with memory impairment (DWRT < 4) and poor cognitive function (MMSE < 25). XGBoost constructs a series of decision trees sequentially, where each tree focuses on correcting the errors of the previous one. This iterative approach refines the model’s predictive accuracy. To enhance the interpretability of the XGBoost model, we used SHapley Additive exPlanations (SHAP) values, which quantify the contribution of each feature to the model’s output. Positive SHAP values indicate a feature’s positive influence on the prediction, whereas negative SHAP values indicate a negative impact [18]. SHAP analyses have been widely used in cognitive research to provide explainable insights into machine learning models [19–21]. We employed Grid Search combined with 5-fold cross-validation to optimize the hyperparameters of the XGBoost model, aiming to balance model performance and minimize overfitting. The target metrics for tuning were Area Under the Curve (AUC) for classification tasks and Root Mean Squared Error (RMSE) for regression tasks. Hyperparameter tuning was performed separately for each outcome variable (i.e., four independent XGBoost models) to ensure that each model was optimized for its specific task. All features used in the adjusted regression models were included in the XGBoost models during the tuning process. We have assessed the predictive performance of each XGBoost model using appropriate metrics, including AUC for classification tasks and RMSE for regression tasks, based on a test set. Detailed results were provided in Supplementary Table 1. To optimize performance and reduce overfitting, the learning rate was adjusted to balance model complexity and generalization, the maximum tree depth was set to control overfitting, and the number of trees was selected to ensure sufficient model capacity. Additionally, hyperparameters were tuned and selected for the proportion of data sampled to train each tree within XGBoost (subsample) and the proportion of features used to train each tree (colsample_bytree).

We also calculated P-values for interaction to assess whether the associations between the composite social isolation score and cognitive outcomes were modified by any of the covariates in the stratified analyses. β coefficients and ORs were reported for each subgroup, along with their corresponding P-values and P-values for interaction, indicating whether significant interactions were present. All statistical analyses were conducted using R software version 4.2.0 (http://www.R-project.org, The R Foundation, Vienna, Austria), with statistical significance set at a two-sided p-value of < 0.05.

## Results

### Demographic characteristics

This study included 25,981 participants, with a mean (SD) age of 62.0 (7.0) years, of whom 28.4% were men. Of them, 42.8% had a primary education or below, 60.6% had manual occupation, and 33.6% reported an annual income of less than 10,000 CNY. Additionally, 80.3% had never smoked, and 68.1% had never consumed alcohol. Participants with higher levels of social isolation tended to be older, had lower educational levels and incomes, and showed a higher prevalence of smoking and alcohol consumption (P-values from < 0.001 to 0.04) (Table 1).

**Table 1:** Characteristics of participants in the Guangzhou biobank cohort study

Characteristics	Total (*N* = 25981)	Composite social isolation score (range 0–7)			*P* value
		0 (*N* = 9250)	1 (*N* = 10874)	≥ 2 (*N* = 5857)	
**Age**,** years**					< 0.001
< 65	16,791 (64.6)	5657 (61.2)	7515 (69.1)	3619 (61.8)	
≥ 65	9190 (35.4)	3593 (38.8)	3359 (30.9)	2238 (38.2)	
**Sex**,** %**					< 0.001
Men	7373 (28.4)	2221 (24.0)	3136 (28.8)	2016 (34.4)	
Women	18,608 (71.6)	7029 (76.0)	7738 (71.2)	3841 (65.6)	
**Education level**,** %**					< 0.001
Primary or below	11,115 (42.8)	3695 (39.9)	4416 (40.6)	3004 (51.3)	
Secondary	12,493 (48.1)	4568 (49.4)	5497 (50.6)	2428 (41.5)	
College or above	2373 (9.1)	987 (10.7)	961 (8.8)	425 (7.2)	
**Occupation**,** %**					< 0.001
Manual	15,733 (60.6)	5354 (57.9)	6531 (60.1)	3848 (65.7)	
Non-manual	6290 (24.2)	2715 (29.4)	2425 (22.3)	1150 (19.6)	
Other	3958 (15.2)	1181 (12.7)	1918 (17.6)	859 (14.7)	
**Personal annual income**,** %**					< 0.001
< 10,000 CNY	8736 (33.6)	3021 (32.7)	3600 (33.1)	2115 (36.1)	
10,000–14,999 CNY	11,223 (43.2)	4042 (43.7)	4768 (43.8)	2413 (41.2)	
≥ 15,000 CNY	4804 (18.5)	1784 (19.3)	2025 (18.6)	995 (17.0)	
Not reported	1218 (4.7)	403 (4.3)	481 (4.5)	334 (5.7)	
**Smoking**,** %**					< 0.001
Never	20,862 (80.3)	7792 (84.2)	8688 (79.9)	4382 (74.8)	
Former	2438 (9.4)	786 (8.5)	999 (9.2)	653 (11.2)	
Current	2681 (10.3)	672 (7.3)	1187 (10.9)	822 (14.0)	
**Sleep duration**,** hours**	7.0 (6.0, 8.0)	7.0 (6.0, 8.0)	7.0 (6.0, 8.0)	7.0 (6.0, 8.0)	< 0.001
**BMI**,** kg/m**^**2**^					0.001
< 18.5	1141 (4.3)	387 (4.2)	479 (4.4)	275 (4.7)	
18.5–24.9	16,153 (62.1)	5698 (61.6)	6788 (62.4)	3667 (62.6)	
25.0–27.4	5431 (20.9)	1952 (21.1)	2260 (20.8)	1219 (20.8)	
≥ 27.5	3256 (12.7)	1213 (13.1)	1347 (12.4)	696 (11.9)	
**Alcohol use**,** %**					< 0.001
Never	17,692 (68.1)	6640 (71.8)	7382 (67.9)	3670 (62.7)	
Former	647 (2.5)	195 (2.1)	266 (2.4)	186 (3.1)	
Current	7642 (29.4)	2415 (26.1)	3226 (29.7)	2001 (34.2)	
**Physical activity**,** %**					< 0.001
Inactive	1937 (7.4)	396 (4.3)	1038 (9.6)	503 (8.6)	
Moderate	10,981 (42.3)	3459 (37.4)	4919 (45.2)	2603 (44.4)	
Active	13,063 (50.3)	5395 (58.3)	4917 (45.2)	2751 (47.0)	
**Self-rated health**,** %**					< 0.001
Good/very good	21,561 (83.0)	7923 (85.7)	9072 (83.4)	4566 (78.0)	
Poor/very poor	4420 (17.0)	1327 (14.3)	1802 (16.6)	1291 (22.0)	
**Self-reported diabetes**,** %**					0.03
No	23,905 (92.0)	8461 (91.5)	10,058 (92.5)	5386 (92.0)	
Yes	2076 (8.0)	789 (8.5)	816 (7.5)	471 (8.0)	
**Self-reported hypertension**,** %**					0.001
No	18,672 (71.9)	6524 (70.5)	7929 (72.9)	4219 (72.0)	
Yes	7309 (28.1)	2726 (29.5)	2945 (27.1)	1638 (28.0)	
**Self-reported dyslipidaemia**,** %**					0.04
No	23,245 (89.5)	8334 (90.1)	9689 (89.1)	5222 (89.2)	
Yes	2736 (10.5)	916 (9.9)	1185 (10.9)	635 (10.8)	

### Association of composite social isolation score with memory decline and cognitive function

Higher levels of social isolation were significantly associated with both memory decline and poorer cognitive function (Tables 2 and 3). After adjustment for potential confounders, each additional point in the composite social isolation score was associated with a lower DWRT score, observed at -0.08 points (95% CI: -0.10 to -0.06). Among varying levels of social isolation, participants with mild social isolation had DWRT scores that were 0.08 points lower (95% CI: -0.13 to -0.03), and those with moderate to high social isolation showed a further lower in scores by 0.15 points (95% CI: -0.21 to -0.09). The statistically significant trend (*P* < 0.001) indicates a consistent pattern where higher social isolation associated with lower DWRT scores (Table 2). Similarly, greater social isolation was associated with lower MMSE scores (β = −0.15; 95% CI: −0.21 to − 0.10) in the adjusted model. Participant with mild social isolation did not show significant changes in MMSE scores (β = -0.05; 95% CI: -0.18 to 0.08), while those with moderate to high social isolation exhibited lower MMSE scores by 0.34 points (95% CI: -0.48 to -0.19) (P for trend < 0.001) (Table 3). Compared to participants with no social isolation, those with moderate to high social isolation had a 27% higher odds of memory impairment (OR = 1.27; 95% CI: 1.15 to 1.40) and a 56% higher odds of poor cognitive function (OR = 1.56; 95% CI: 1.23 to 1.99) (Tables 2 and 3).

**Table 2:** Association of composite social isolation score with delayed word recall test scores (DWRT scores) and memory impairment in Guangzhou biobank cohort study (*N* = 25981)

	DWRT scores, β (95% CI)		Memory impairment, OR (95% CI)	
	Crude model	Adjusted model ^a^	Crude model	Adjusted model ^a^
**Composite social isolation score, per point**	-0.13 (-0.15, -0.10) ^***^	-0.08 (-0.10, -0.06) ^*^	1.19 (1.14, 1.23) ^***^	1.12 (1.08, 1.17) ^**^
**Composite social isolation score group**				
No social isolation	0.00	0.00	1.00	1.00
Mild social isolation	-0.05 (-0.10, -0.001) ^*^	-0.08 (-0.13, -0.03) ^**^	1.15 (1.05, 1.25) ^**^	1.19 (1.09, 1.30) ^**^
Moderate to high social isolation	-0.26 (-0.32, -0.21) ^***^	-0.15 (-0.21, -0.09) ^**^	1.44 (1.31, 1.58) ^***^	1.27 (1.15, 1.40) ^***^
P for trend	< 0.001	< 0.001	0.001	< 0.001

DWRT = Delayed 10-word Recall Test; Memory impairment: DWRT scores < 4; OR: odds ratio; CI: confidence interval. No social isolation: composite social isolation score = 0; mild social isolation: 1; moderate to high social isolation: ≥2

^a^: Adjusted for age, sex, education level, occupation, personal annual income, smoking, sleep duration, BMI, alcohol use, physical activity, self-rated health, self-reported diabetes, self-reported hypertension, self-reported dyslipidaemia

*: *P* < 0.05; **: *P* < 0.01; ***: *P* < 0.001

**Table 3:** Association of composite social isolation score with mini-mental state examination scores (MMSE scores) and cognitive function in Guangzhou biobank cohort study (*N* = 6815)

	MMSE scores, β (95% CI)		Poor cognitive function, OR (95% CI)	
	Crude model	Adjusted model ^a^	Crude model	Adjusted model ^a^
**Composite social isolation score, per point**	-0.34 (-0.40, -0.27) ***	-0.15 (-0.21, -0.10) ***	1.40 (1.30, 1.51) ***	1.19 (1.09, 1.30) ***
**Composite social isolation score group**				
No social isolation	0.00	0.00	1.00	1.00
Mild social isolation	-0.12 (-0.27, 0.02)	-0.05 (-0.18, 0.08)	1.30 (1.04, 1.62) ***	1.22 (0.97, 1.56) ***
Moderate to high social isolation	-0.78 (-0.94, -0.62) ***	-0.34 (-0.48, -0.19) ***	2.33 (1.88, 2.91) ***	1.56 (1.23,1.99) ***
P for trend	< 0.001	< 0.001	< 0.001	< 0.001

MMSE = Mini-Mental State Examination; Poor cognitive function: MMSE scores < 25; OR: odds ratio; CI: confidence interval. No social isolation: composite social isolation score = 0; mild social isolation: 1; moderate to high social isolation: ≥2

^a^: Adjusted for age, sex, education level, occupation, personal annual income, smoking, sleep duration, BMI, alcohol use, physical activity, self-rated health, self-reported diabetes, self-reported hypertension, self-reported dyslipidaemia

^*^: *P* < 0.05; ^**^: *P* < 0.01; ^***^: *P* < 0.001

### XGBoost algorithm models reveal relative importance in memory decline and cognitive function

**Fig. 1:**
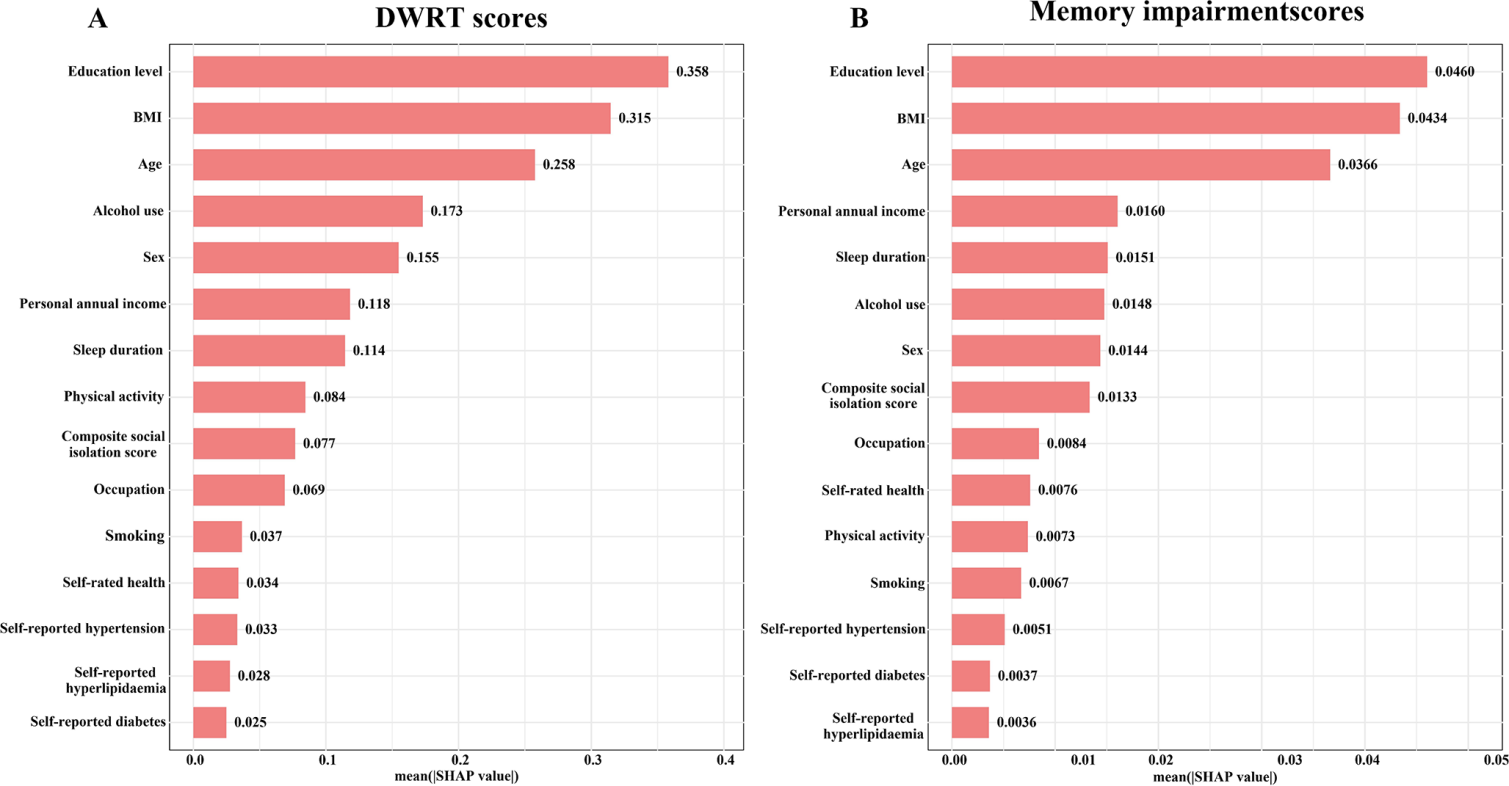
Contribution of socio-demographic and health factors to DWRT Score (**A**) s and memory impairment (**B**) as assessed by SHAP values. Note: All models were adjusted for age, sex, education level, occupation, personal annual income, smoking, sleep duration, BMI, alcohol use, physical activity, self-rated health, self-reported diabetes, self-reported hypertension, self-reported dyslipidaemia

**Fig. 2:**
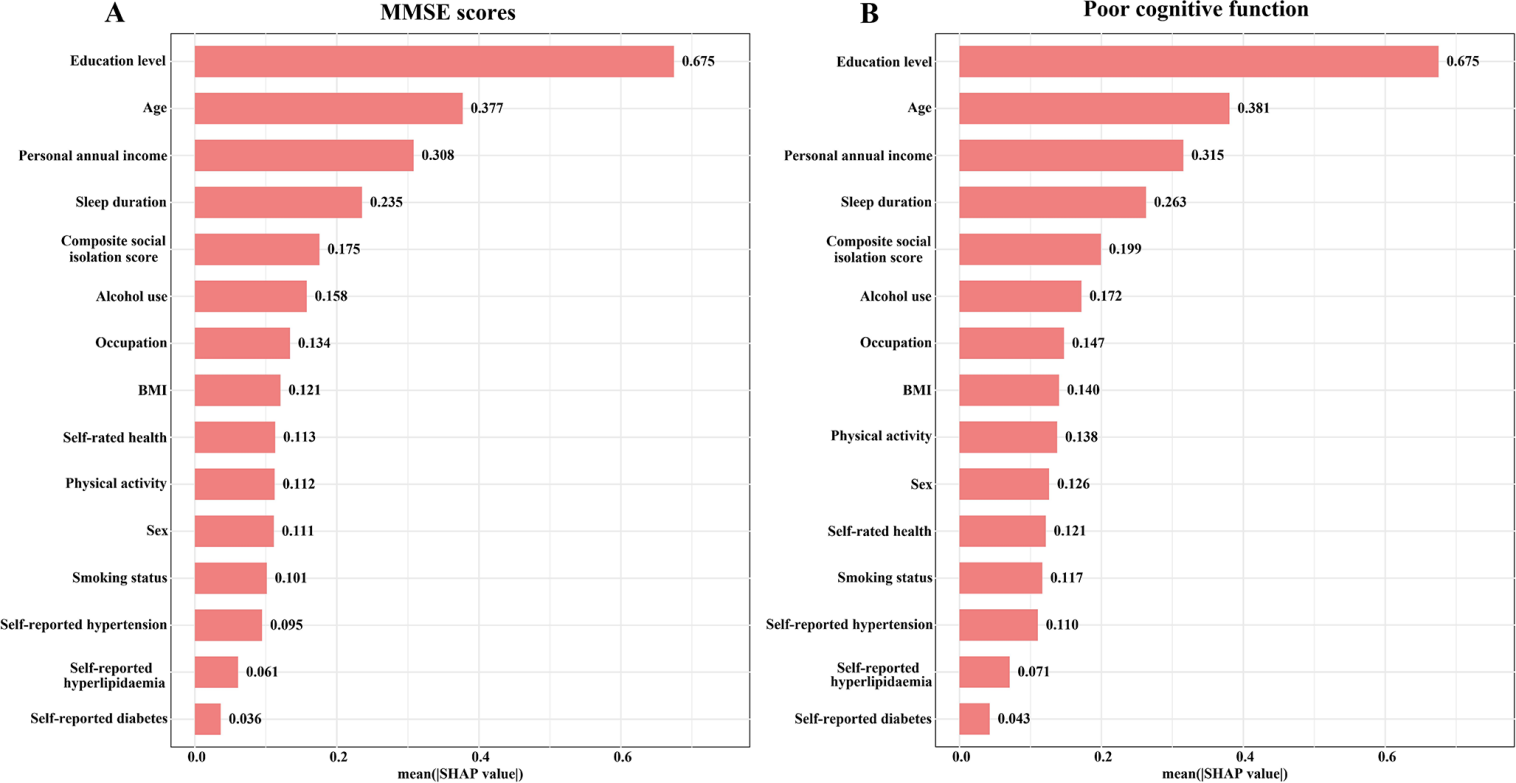
Contribution of socio-demographic and health factors to MMSE Score (**A**) s and poor cognitive function (**B**) as assessed by SHAP values. Note: All models were adjusted for age, sex, education level, occupation, personal annual income, smoking, sleep duration, BMI, alcohol use, physical activity, self-rated health, self-reported diabetes, self-reported hypertension, self-reported dyslipidaemia

In terms of feature importance as determined by SHAP values, social isolation was ranked ninth in its association with DWRT scores, with a SHAP value of 0.077 (Fig. 1A). It was ranked eighth for its association with memory impairment, with a SHAP value of 0.0133 (Fig. 1B). Although the associations of social isolation were less pronounced than those of education, BMI, and age, it was a more important predictor than smoking status, occupation, and self-reported health. In MMSE predictions (Fig. 2A), social isolation ranked fifth (SHAP value = 0.175) and similarly for poor cognitive function (Fig. 2B), with a SHAP value of 0.199.

### Subgroup analysis

Stratified analyses showed that the impact of social isolation on cognitive outcomes, including both DWRT and MMSE scores, was stronger in certain subgroups (Tables 4 and 5). In older adults (≥ 65 years), the associations with DWRT scores (β = −0.18; 95% CI: −0.22 to − 0.14) and MMSE scores (β = −0.46; 95% CI: −0.60 to − 0.32) were more pronounced, as well as a higher odds of memory impairment (OR = 1.21; 95% CI: 1.15 to 1.27) and poor cognitive function (OR = 1.28; 95% CI: 1.15 to 1.42), compared to younger individuals. Participants with primary education or below showed stronger associations with both DWRT (β = −0.13; 95% CI: −0.17 to − 0.10) and MMSE scores (β = −0.40; 95% CI: −0.53 to − 0.27), as well as a higher risk of memory impairment (OR = 1.14; 95% CI: 1.08 to 1.19) and poor cognitive function (OR = 1.27; 95% CI: 1.15 to 1.39). Those with manual occupation also showed more pronounced negative associations with both DWRT (β = −0.14; 95% CI: −0.17 to − 0.11) and MMSE scores (β = −0.36; 95% CI: −0.45 to − 0.28), and a higher risk of cognitive decline. Additionally, the associations also appear to be stronger in those with lower income, BMI (< 18.5 kg/m²), and poor physical activity (Tables 4 and 5).

**Table 4:** Stratified analyses to identify variables that may modify the association of composite social isolation score with delayed word recall test scores (DWRT scores) and memory impairment

	DWRT scores			Memory impairment		
	β (95% CI)	*P*-value	*P*-value for interaction	OR (95% CI)	*P*-value	*P*-value for interaction
**Age**,** %**			< 0.001			0.32
< 65	-0.09 (-0.12, -0.06) ***	< 0.001		1.16 (1.1, 1.23) ***	< 0.001	
≥ 65	-0.18 (-0.22, -0.14) ***	< 0.001		1.21 (1.15, 1.27) ***	< 0.001	
**Sex**,** %**			0.31			0.92
Men	-0.1 (-0.14, -0.06) ***	< 0.001		1.18 (1.1, 1.27) ***	< 0.001	
Women	-0.13 (-0.16, -0.1) ***	< 0.001		1.19 (1.13, 1.24) ***	< 0.001	
**Education level**,** %**			< 0.001			0.67
Primary or below	-0.13 (-0.17, -0.1) ***	< 0.001		1.14 (1.08, 1.19) ***	< 0.001	
Secondary	-0.04 (-0.08, -0.01) *	0.02		1.14 (1.06, 1.23) ***	< 0.001	
College or above	-0.02 (-0.1, 0.07)	0.69		1.04 (0.87, 1.26)	0.65	
**Occupation**,** %**			0.002			0.15
Manual	-0.14 (-0.17, -0.11) ***	< 0.001		1.18 (1.12, 1.23) ***	< 0.001	
Non-manual	-0.03 (-0.08, 0.02)	0.29		1.07 (0.97, 1.19)	0.16	
Other	-0.11 (-0.18, -0.05) **	0.001		1.23 (1.1, 1.37) ***	< 0.001	
**Personal annual income**,** %**			0.04			0.68
< 10,000	-0.16 (-0.2, -0.12) ***	< 0.001		1.18 (1.11, 1.26) ***	< 0.001	
10,000–14,999	-0.11 (-0.15, -0.07) ***	< 0.001		1.18 (1.11, 1.26) ***	< 0.001	
≥ 15,000	-0.08 (-0.14, -0.03) *	0.01		1.18 (1.06, 1.32) **	0.003	
Not reported	-0.03 (-0.13, 0.07)	0.57		1.08 (0.93, 1.24)	0.321	
**Smoking**,** %**			0.86			0.69
Never	-0.12 (-0.15, -0.09) ***	< 0.001		1.18 (1.13, 1.23) ***	< 0.001	
Former	-0.10 (-0.17, -0.03) *	0.01		1.14 (1.02, 1.28) *	0.03	
Current	-0.11 (-0.19, -0.04) **	0.001		1.22 (1.09, 1.37) ***	< 0.001	
**BMI**,** kg/m**^**2**^, **%**			0.05			0.08
< 18.5	-0.19 (-0.31, -0.08) **	0.001		1.15 (0.97, 1.36)	0.11	
18.5–24.9	-0.14 (-0.17, -0.11) ***	< 0.001		1.23 (1.17, 1.3) ***	< 0.001	
25.0–27.4	-0.12 (-0.17, -0.06) ***	< 0.001		1.15 (1.05, 1.25) **	0.002	
≥ 27.5	-0.04 (-0.11, 0.02)	0.21		1.07 (0.96, 1.19)	0.25	
**Alcohol use**,** %**			0.73			0.89
Never	-0.15 (-0.18, -0.12) ***	< 0.001		1.21 (1.16, 1.27) ***	< 0.001	
Former	-0.14 (-0.29, 0)	0.05		1.16 (0.95, 1.42)	0.14	
Current	-0.13 (-0.18, -0.09) ***	< 0.001		1.2 (1.11, 1.29) ***	< 0.001	
**Physical activity**,** %**			0.001			0.04
Inactive	-0.20 (-0.29, -0.12) ***	< 0.001		1.35 (1.17, 1.56) ***	< 0.001	
Moderate	-0.16 (-0.2, -0.12) ***	< 0.001		1.22 (1.15, 1.29) ***	< 0.001	
Active	-0.07 (-0.11, -0.04) ***	< 0.001		1.13 (1.07, 1.2) ***	< 0.001	
**Self-rated health**,** %**			0.01			0.52
Good/very good	-0.11 (-0.13, -0.08) ***	< 0.001		1.17 (1.12, 1.22) ***	< 0.001	
Poor/very poor	-0.19 (-0.25, -0.14) ***	< 0.001		1.2 (1.11, 1.3) ***	< 0.001	

**Table 5:** Stratified analyses to identify variables that may modify the association of composite social isolation score with mini-mental state examination scores (MMSE scores) and cognitive function

	MMSE scores			Poor cognitive function		
	β (95% CI)	*P*-value	*P*-value for interaction	OR (95% CI)	*P*-value	*P*-value for interaction
**Age**,** %**			< 0.001			0.29
< 65	-0.22 (-0.29, -0.16) ***	< 0.001		1.43 (1.28, 1.59) ***	< 0.001	
≥ 65	-0.46 (-0.6, -0.32) ***	< 0.001		1.28 (1.15, 1.42) ***	< 0.001	
**Sex**,** %**			0.15			0.13
Men	-0.34 (-0.45, -0.23) ***	< 0.001		1.39 (1.2, 1.6) ***	< 0.001	
Women	-0.36 (-0.43, -0.28) ***	< 0.001		1.44 (1.31, 1.58) ***	< 0.001	
**Education level**,** %**			< 0.001			0.27
Primary or below	-0.4 (-0.53, -0.27) ***	< 0.001		1.27 (1.15, 1.39) ***	< 0.001	
Secondary	-0.09 (-0.15, -0.03) **	0.004		1.24 (1.04, 1.47) *	0.017	
College or above	-0.08 (-0.19, 0.02)	0.134		0.76 (0.27, 2.17)	0.608	
**Occupation**,** %**			0.73			0.77
Manual	-0.36 (-0.45, -0.28) ***	< 0.001		1.38 (1.26, 1.5) ***	< 0.001	
Non-manual	-0.13 (-0.22, -0.03) **	0.008		1.24 (0.95, 1.63)	0.12	
Other	-0.36 (-0.51, -0.21) ***	< 0.001		1.49 (1.21, 1.84) ***	< 0.001	
**Personal annual income**,** %**			0.53			0.16
< 10,000	-0.5 (-0.65, -0.34) ***	< 0.001		1.39 (1.23, 1.56) ***	< 0.001	
10,000–14,999	-0.17 (-0.25, -0.09) ***	< 0.001		1.21 (1.05, 1.38) **	0.007	
≥ 15,000	-0.17 (-0.26, -0.08) ***	< 0.001		1.51 (1.19, 1.9) **	0.001	
Not reported	-0.4 (-0.76, -0.03) *	0.033		1.36 (1.02, 1.8) *	0.037	
**Smoking**,** %**			0.71			0.18
Never	-0.35 (-0.42, -0.28) ***	< 0.001		1.46 (1.34, 1.6) ***	< 0.001	
Former	-0.12 (-0.3, 0.07)	0.211		1.08 (0.86, 1.37)	0.495	
Current	-0.37 (-0.58, -0.17) ***	< 0.001		1.29 (1.04, 1.58) *	0.018	
**BMI**,** kg/m**^**2**^, **%**			0.24			0.87
< 18.5	-0.47 (-0.89, -0.06) *	0.025		1.67 (1.21, 2.3) **	0.002	
18.5–24.9	-0.32 (-0.4, -0.24) ***	< 0.001		1.39 (1.26, 1.54) ***	< 0.001	
25.0–27.4	-0.33 (-0.46, -0.2) ***	< 0.001		1.37 (1.16, 1.63) ***	< 0.001	
≥ 27.5	-0.38 (-0.55, -0.2) ***	< 0.001		1.37 (1.12, 1.69) **	0.003	
**Alcohol use**,** %**			< 0.001			0.07
Never	-0.57 (-0.7, -0.45) ***	< 0.001		1.55 (1.38, 1.75) ***	< 0.001	
Former	-0.21 (-0.54, 0.11)	0.202		0.89 (0.6, 1.3)	0.541	
Current	-0.22 (-0.29, -0.15) ***	< 0.001		1.35 (1.21, 1.5) ***	< 0.001	
**Physical activity**,** %**			0.94			0.63
Inactive	-0.54 (-0.81, -0.26) ***	< 0.001		1.42 (1.06, 1.91) *	0.02	
Moderate	-0.33 (-0.47, -0.2) ***	< 0.001		1.38 (1.19, 1.61) ***	< 0.001	
Active	-0.31 (-0.39, -0.24) ***	< 0.001		1.4 (1.27, 1.53) ***	< 0.001	
**Self-rated health**,** %**			0.51			0.86
Good/very good	-0.36 (-0.51, -0.21) ***	< 0.001		1.35 (1.17, 1.55) ***	< 0.001	
Poor/very poor	-0.3 (-0.37, -0.23) ***	< 0.001		1.38 (1.26, 1.52) ***	< 0.001	

## Discussion

Our study rigorously quantified the associations between social isolation and cognitive decline within a large cohort from older Chinese adults, addressing a notable gap in the existing literature on the relative importance of social isolation in the context of other established cognitive risk factors. Despite the known risk factors such as education, age, and BMI, our findings elucidate the significant role that social isolation plays in cognitive health, ranking as a substantive factor in both the DWRT and the MMSE scores. Particularly, the XGBoost analysis indicated social isolation as a non-negligible predictor, highlighting its relevance amidst more traditionally recognized determinants of cognitive impairment. This study emphasizes the need for public health strategies that not only address typical biomedical and lifestyle factors but also consider the cognitive risks associated with social isolation, especially in aging populations.

Social isolation contributes to cognitive impairment through multiple pathways. Epidemiological studies consistently show that reduced social interaction reduces cognitive stimulation, which is essential for maintaining cognitive health, particularly in older adults. This reduction undermines cognitive reserve, which is fostered through engaging social activities and acts as a protective buffer against cognitive deterioration [22]. Moreover, individuals who are socially isolated often experience increased psychological stress and reduced emotional support, both well-established risk factors for cognitive decline [23]. Associated unhealthy behaviors, including inadequate nutrition, physical inactivity, worse sleep, smoking, and excessive alcohol use, are prevalent in those who are socially isolated. These behaviors not only contribute to the acceleration of neurodegenerative processes but also diminish overall brain health, perpetuating a cycle of adverse health outcomes that further impair both physical and cognitive functions [24–28]. Additionally, social isolation induces chronic stress that disrupts the hypothalamic-pituitary-adrenal (HPA) axis, leading to elevated cortisol levels [15]. Such prolonged exposure to cortisol is linked to hippocampal atrophy, which significantly impairs memory and cognitive functions. This dysregulation also compromises the stress response mechanisms, accelerating cognitive decline, especially in neurodegenerative conditions like Alzheimer’s disease [29]. Furthermore, social isolation is associated with increased systemic inflammation, which accelerates neurodegeneration and cognitive impairment [12]. These physiological and behavioral changes collectively exacerbate memory decline and overall cognitive performance. Given the profound implications for public health, addressing social isolation is imperative for preserving cognitive health, especially in older adults [14].

In our study, older adults (≥ 65 years) showed stronger negative associations with both DWRT and MMSE scores, indicating greater susceptibility to memory impairment and cognitive decline. This is consistent with existing epidemiological evidence showing that older adults often encounter additional challenges such as diminished social networks, reduced mobility, and limited access to social resources, all of which exacerbate the cognitive consequences of social isolation [30]. Similarly, participants with lower education, particularly those with only primary education or less, showed more pronounced associations between social isolation and cognitive decline. This aligns with research suggesting that lower education is associated with reduced cognitive reserve, increasing vulnerability to cognitive deterioration when exposed to adverse factors such as social isolation [7]. Moreover, individuals with manual occupation, typically characterized by lower socioeconomic status and fewer opportunities for cognitive engagement, experienced greater negative associations between social isolation and cognitive outcomes [31]. These findings highlight the importance of addressing social isolation, especially in older adults and socioeconomically disadvantaged groups, to mitigate its detrimental effects on cognitive health. Tailored interventions that address the unique challenges faced by these vulnerable populations are crucial in preserving cognitive function and reducing health disparities associated with social isolation.

Interventions to reduce social isolation are crucial for preserving cognitive health, especially among aging populations. Epidemiological evidence shows that community-based activities, social support groups, and virtual platforms are highly effective in fostering social engagement, thereby reducing the risk of cognitive decline [24]. Furthermore, promoting physical activity, mental health support, and healthy behaviors like proper diet has been shown to enhance neuroplasticity and protect against brain deterioration [32]. Targeting these interventions toward vulnerable groups—such as older adults, those with lower socioeconomic position (lower education or manual occupation)—who often face greater isolation due to mobility limitations and restricted access to social resources, is essential for improving cognitive outcomes. Additionally, public health policies must address the broader social determinants, including socioeconomic disparities and access to education, which exacerbate social isolation and contribute to cognitive decline [33].

There are several limitations to this study that should be acknowledged. First, the cross-sectional design of the study limits the ability to draw causal inferences about the association between social isolation and cognitive decline. Further studies using longitudinal design are required to establish temporal relationships and to investigate the potential long-term effects of social isolation on cognitive outcomes. Second, while we controlled for a range of confounders, there may be unmeasured variables that could influence the observed associations. For example, the quality of social interactions, which may be as important as the quantity, was not assessed in this study.

Third, an additional limitation of this study is the age of the data, collected between 2003 and 2006. Since then, China has experienced rapid socioeconomic development, including improvements in living standards, economic prosperity, and urbanization. These changes may have affected the prevalence of social isolation and its health impacts, limiting the generalizability of our findings to present-day China. A key strength of this study is its focus on the GBCS, offering valuable insights into the effects of social isolation within a unique cultural and socioeconomic context. Additionally, the social isolation score was specifically developed based on this cohort, ensuring that the measure is highly relevant and tailored to the participants. These factors enhance the validity of the findings and provide a strong foundation for future comparative studies across diverse populations to assess whether similar patterns of social isolation and cognitive decline are observed.

## Conclusion

This study addresses the gap in understanding the relative importance of social isolation as a predictor of cognitive decline. Our findings showed that social isolation, alongside established factors like education and age, was significantly associated with memory impairment and poor cognitive function, particularly in vulnerable populations. These results emphasize the need for targeted public health interventions to reduce social isolation and support cognitive health, especially among at-risk groups such as older adults and individuals with lower socioeconomic status.

## Electronic supplementary material

Below is the link to the electronic supplementary material.

Supplementary Material 1

## Data Availability

Ethical approval permits us to share data upon request. Please direct any such requests to the Guangzhou Biobank Cohort Study Data Access Committee at gbcsdata@hku.hk.

## References

[1] Pais R, Ruano L, O PC, Barros H (2020) Global cognitive impairment prevalence and incidence in community dwelling older Adults-A systematic review. Geriatr (Basel) 5(4) 10.3390/geriatrics5040084 PMC7709591 33121002

[2] Bai W, Chen P, Cai H, Zhang Q, Su Z, Cheung T, Jackson T, Sha S, Xiang YT (2022) Worldwide prevalence of mild cognitive impairment among community dwellers aged 50 years and older: a meta-analysis and systematic review of epidemiology studies. Age Ageing 51(8) 10.1093/ageing/afac173 35977150

[3] Shankar A, Hamer M, McMunn A, Steptoe A (2013) Social isolation and loneliness: relationships with cognitive function during 4 years of follow-up in the english longitudinal study of ageing. Psychosom Med 75(2):161–170 23362501 10.1097/PSY.0b013e31827f09cd

[4] Evans IEM, Martyr A, Collins R, Brayne C, Clare L (2019) Social isolation and cognitive function in later life: A systematic review and Meta-Analysis. J Alzheimers Dis 70(s1):S119–s144 30372678 10.3233/JAD-180501 PMC6700717

[5] DiNapoli EA, Wu B, Scogin F (2014) Social isolation and cognitive function in Appalachian older adults. Res Aging 36(2):161–179 25650688 10.1177/0164027512470704

[6] Fakoya OA, McCorry NK, Donnelly M (2020) Loneliness and social isolation interventions for older adults: a scoping review of reviews. BMC Public Health 20(1):129 32054474 10.1186/s12889-020-8251-6 PMC7020371

[7] Piolatto M, Bianchi F, Rota M, Marengoni A, Akbaritabar A, Squazzoni F (2022) The effect of social relationships on cognitive decline in older adults: an updated systematic review and meta-analysis of longitudinal cohort studies. BMC Public Health 22(1):278 35148704 10.1186/s12889-022-12567-5 PMC8831686

[8] Jiang C, Thomas GN, Lam TH, Schooling CM, Zhang W, Lao X, Adab P, Liu B, Leung GM, Cheng KK (2006) Cohort profile: the Guangzhou biobank cohort study, a Guangzhou-Hong Kong-Birmingham collaboration. Int J Epidemiol 35(4):844–852 16844769 10.1093/ije/dyl131

[9] Berkman LF, Syme SL (1979) Social networks, host resistance, and mortality: a nine-year follow-up study of Alameda County residents. Am J Epidemiol 109(2):186–204 425958 10.1093/oxfordjournals.aje.a112674

[10] Wang J, Zhang WS, Jiang CQ, Zhu F, Jin YL, Cheng KK, Lam TH, Xu L (2022) Associations of face-to-face and non-face-to-face social isolation with all-cause and cause-specific mortality: 13-year follow-up of the Guangzhou biobank cohort study. BMC Med 20(1):178 35501792 10.1186/s12916-022-02368-3 PMC9059436

[11] Folstein MF, Folstein SE, McHugh PR (1975) Mini-mental State. A practical method for grading the cognitive State.of patients for the clinician. J Psychiatr Res 12(3):189–198 1202204 10.1016/0022-3956(75)90026-6

[12] Knopman DS, Ryberg S (1989) A verbal memory test with high predictive accuracy for dementia of the alzheimer type. Arch Neurol 46(2):141–145 2916953 10.1001/archneur.1989.00520380041011

[13] Tian YM, Zhang WS, Jiang CQ, Zhu F, Jin YL, Zhu T, Cheng KK, Xu L (2022) Association of alcohol use with memory decline in middle-aged and older Chinese: a longitudinal cohort study. BMC Psychiatry 22(1):673 36320000 10.1186/s12888-022-04298-z PMC9623936

[14] Welsh KA, Butters N, Mohs RC, Beekly D, Edland S, Fillenbaum G, Heyman A (1994) The consortium to Establish a registry for Alzheimer’s disease (CERAD). Part V. A normative study of the neuropsychological battery. Neurology 44(4):609–614 8164812 10.1212/wnl.44.4.609

[15] Xu L, Jiang CQ, Lam TH, Zhang WS, Cherny SS, Thomas GN, Cheng KK (2014) Sleep duration and memory in the elderly Chinese: longitudinal analysis of the Guangzhou biobank cohort study. Sleep 37(11):1737–1744 25364069 10.5665/sleep.4162 PMC4196057

[16] Ojala AK, Schalin-Jäntti C, Pitkälä KH, Tilvis RS, Strandberg TE (2016) Serum thyroid-stimulating hormone and cognition in older people. Age Ageing 45(1):155–157 26601696 10.1093/ageing/afv160

[17] Chen T, Guestrin C (2016) XGBoost: A Scalable Tree Boosting System. In: Proceedings of the 22nd ACM SIGKDD International Conference on Knowledge Discovery and Data Mining. San Francisco, California, USA: Association for Computing Machinery;: 785–794

[18] Lundberg SM, Lee SI (2017) A Unified Approach to Interpreting Model Predictions. Adv Neural Inf Process Syst 30:4765–4774.

[19] Sakal C, Li T, Li J, Li X (2024) Predicting poor performance on cognitive tests among older adults using wearable device data and machine learning: a feasibility study. NPJ Aging 10(1):56 39587119 10.1038/s41514-024-00177-x PMC11589133

[20] You J, Zhang YR, Wang HF, Yang M, Feng JF, Yu JT, Cheng W (2022) Development of a novel dementia risk prediction model in the general population: A large, longitudinal, population-based machine-learning study. EClinicalMedicine 53:101665 36187723 10.1016/j.eclinm.2022.101665 PMC9519470

[21] Zhang X, Liao Y, Zhang D, Liu W, Wang Z, Jin Y, Chen S, Wei J (2025) Explainable machine learning models for identifying mild cognitive impairment in older patients with chronic pain. BMC Nurs 24(1):72 39838390 10.1186/s12912-025-02723-8 PMC11748277

[22] Liang YY, Chen Y, Feng H, Liu X, Ai QH, Xue H, Shu X, Weng F, He Z, Ma J et al (2023) Association of social isolation and loneliness with incident heart failure in a Population-Based cohort study. JACC Heart Fail 11(3):334–344 36737310 10.1016/j.jchf.2022.11.028 PMC9891238

[23] Klinenberg E (2016) Social isolation, loneliness, and living alone: identifying the risks for public health. Am J Public Health 106(5):786–787 27049414 10.2105/AJPH.2016.303166 PMC4985072

[24] Xian G, Chai Y, Gong Y, He W, Ma C, Zhang X, Zhang J, Ma Y (2024) The relationship between healthy lifestyles and cognitive function in Chinese older adults: the mediating effect of depressive symptoms. BMC Geriatr 24(1):299 38549104 10.1186/s12877-024-04922-5 PMC10979595

[25] Yu B, Steptoe A, Niu K, Ku PW, Chen LJ (2018) Prospective associations of social isolation and loneliness with poor sleep quality in older adults. Qual Life Res 27(3):683–691 29188485 10.1007/s11136-017-1752-9

[26] Rosenau C, Köhler S, Soons LM, Anstey KJ, Brayne C, Brodaty H, Engedal K, Farina FR, Ganguli M, Livingston G et al (2024) Umbrella review and Delphi study on modifiable factors for dementia risk reduction. Alzheimers Dement 20(3):2223–2239 38159267 10.1002/alz.13577 PMC10984497

[27] Sakal C, Li T, Li J, Yang C, Li X (2024) Association between sleep efficiency variability and cognition among older adults: Cross-Sectional accelerometer study. JMIR Aging 7:e54353 38596863 10.2196/54353 PMC11007383

[28] Bloomberg M, Brocklebank L, Doherty A, Hamer M, Steptoe A (2024) Associations of accelerometer-measured physical activity, sedentary behaviour, and sleep with next-day cognitive performance in older adults: a micro-longitudinal study. Int J Behav Nutr Phys Act 21(1):133 39654035 10.1186/s12966-024-01683-7 PMC11629534

[29] O’Connor TM, O’Halloran DJ, Shanahan F (2000) The stress response and the hypothalamic-pituitary-adrenal axis: from molecule to melancholia. QJM 93(6):323–333 10873181 10.1093/qjmed/93.6.323

[30] Lee CY, Goldstein SE (2016) Loneliness, stress, and social support in young adulthood: does the source of support matter?? J Youth Adolesc 45(3):568–580 26602564 10.1007/s10964-015-0395-9

[31] Cardona M, Andrés P (2023) Are social isolation and loneliness associated with cognitive decline in ageing? Front Aging Neurosci 15:1075563 36909946 10.3389/fnagi.2023.1075563 PMC9995915

[32] Hämmig O (2019) Health risks associated with social isolation in general and in young, middle and old age. PLoS ONE 14(7):e0219663 31318898 10.1371/journal.pone.0219663 PMC6638933

[33] Ng R, Sutradhar R, Yao Z, Wodchis WP, Rosella LC (2020) Smoking, drinking, diet and physical activity-modifiable lifestyle risk factors and their associations with age to first chronic disease. Int J Epidemiol 49(1):113–130 31329872 10.1093/ije/dyz078 PMC7124486

