# Supplementary material for: Associations of social isolation with memory and cognitive function in middle-aged and older Chinese adults
Source: Aging Clin Exp Res. 2025 Mar 8;37(1):71. doi: 10.1007/s40520-025-02987-9 (PMC11889041; doi:10.1007/s40520-025-02987-9)
Supplement: Supplementary file 1 — Supplementary Material 1 [file 40520_2025_2987_MOESM1_ESM.docx]

**Supplementary Table 1.** Predictive Performance of XGBoost Models

|  | DWRT scores | Memory impairment | MMSE scores | Poor cognitive function |
| --- | --- | --- | --- | --- |
| AUC | - | 0.74 | - | 0.72 |
| RMSE | 1.34 | - | 1.52 | - |

AUC: Area Under the Curve; RMSE: Root Mean Squared Error. DWRT = Delayed 10-word Recall Test; Memory impairment: DWRT scores < 4; MMSE = Mini-Mental State Examination; Poor cognitive function: MMSE scores < 25
